# Three-Dimensional Superhydrophobic Hollow Hemispherical MXene for Efficient Water-in-Oil Emulsions Separation

**DOI:** 10.3390/nano11112866

**Published:** 2021-10-27

**Authors:** Haoran Chen, Riyuan Wang, Weiming Meng, Fanglin Chen, Tao Li, Dingding Wang, Chunxiang Wei, Hongdian Lu, Wei Yang

**Affiliations:** School of Energy, Materials and Chemical Engineering, Hefei University, Hefei 230601, China; CHR1344@163.com (H.C.); YRW1632021@163.com (R.W.); weiming_Meng@163.com (W.M.); cfl1234560628@126.com (F.C.); litao18856962039@163.com (T.L.); DDW1632021@163.com (D.W.); weichunxiang@hfuu.edu.cn (C.W.)

**Keywords:** MXene, polystyrene template, superhydrophobicity, water-in-oil emulsion separation

## Abstract

A superhydrophobic macroporous material composed of hollow hemispherical MXene (HSMX) was synthesized by the thermal annealing of MXene-wrapped cationic polystyrene spheres (CPS@MXene). Notably, the spherical MXene shells exhibited highly efficient catalysis of the carbonization of CPS into carbon nanoparticles. Their insertion into the interlayer of MXene increased the d-spacing and created hollow hemispheres. The as-prepared HSMX with nanoscale walls had a lower packing density than MXene, but higher porosity, total pore volume, and total pore area. Moreover, the stacking of hollow hemispheres promoted the formation of a highly undulating macroporous surface and significantly improved the surface roughness of the HSMX-based 3D membrane, resulting in superhydrophobicity with a water contact angle of 156.4° and a rolling angle of 6°. As a result, the membrane exhibited good separation efficiency and *Flux* for emulsifier-stabilized water-in-paraffin liquid emulsions, which was dependent on its superhydrophobic performance and strong demulsification ability derived from the razor effect originating from the ultrathin walls of HSMX. This work provides a facile approach for the transformation of highly hydrophilic 2D MXene into superhydrophobic 3D HSMX, and opens a new pathway for the development of advanced MXene-based materials for environmental remediation applications.

## 1. Introduction

Recently, a new class of graphene-like two dimensional (2D) crystalline transition-metal carbides (MXenes), mainly in the form of Ti_3_C_2_T_x_-MXene, has shown great prospects in various fields, including energy conversion and storage, and electromagnetic interference shielding, owing to their unique physicochemical properties [1,2,3,4]. In addition, the negatively charged surfaces of MXenes obtained through conventional chemical liquid etching methods are usually terminated with hydrophilic groups, such as –OH, –O, and –F [5,6,7,8], which makes them promising candidate materials for environmental applications, such as oil-in-water separation and pollutant treatment [9,10,11]. For instance, the Ti_3_C_2_T_x_-MXene membrane with good hydrophilicity and underwater superoleophobic performance could achieve highly efficient oil-in-water emulsion separation with excellent separation efficiency of over 99.4% and a high permeation *Flux* of 887 L·m^−2^·h^−1^·bar^−1^ [9].

It is known that 2D nanomaterials, including MXene, graphene, and clay, are prone to restacking when their corresponding nanosheets are peeled off owing to the intrinsic intermolecular forces, such as van der Waals forces and hydrogen bonding, which weaken their nanoscale dispersion and activities [12,13,14,15,16,17,18]. The assembly of 2D nanosheets into sophisticated three-dimensional (3D) macroscopic architectures, such as porous networks and films [19,20,21,22], foams [23], aerogels, and hydrogels [24,25], is an effective approach to overcome the restacking problem, conferring exceptional properties and extending their capability in diverse practical applications.

However, it is difficult to obtain a 3D MXene material directly from individual 2D nanosheets owing to the inherent characteristics of MXene. The template method is a simple and flexible approach to realize controllable and spatially well-defined 3D MXene materials prepared with various diameters, depending on the dimensions of the template used. The typical preparation process involves the wrapping of MXene nanosheets on the template surface followed by the removal of template via thermal annealing or solvent extraction. The commonly used templates include ice [26], poly(methyl methacrylate) (PMMA) [27,28], and polystyrene (PS) spheres [29]. In addition to focusing on the electrochemical performance, studies of 3D MXene materials have shown good environmental characteristics in applications such as the adsorption of heavy metal ions and organic contaminants [30,31] and seawater desalination [32,33], owing to their open porous structure, high specific surface area, and abundance of functional groups on the surface. Moreover, it has been found that the surface wetting behavior of 2D MXene can be adjusted from hydrophilic to hydrophobic by the assembly of 3D MXene [28,34,35,36]. As reported in the literature, a free-standing and flexible 3D macroporous MXene film with a hollow spherical framework prepared via a sacrificial PMMA template exhibited good hydrophobicity with a high water contact angle (WCA) of 135°. Compared with a compact 2D structural MXene film, the greater surface roughness and abundance of pores of 3D MXene are thought to account for the reversal of wetting behavior [28]. It therefore seems to follow that the surface wetting behavior may be improved to superhydrophobicity by regulating the porosity and surface characteristics of 3D MXene, thus contributing to the further application of MXene-based materials toward environmental remediation.

Although 2D MXene materials with outstanding hydrophilicity exhibit great potential as highly efficient separation membranes for oil-in-water emulsions, there are few reports on the construction of superhydrophobic 3D MXene materials, and the various effects on their surface wetting behavior and applications in water-in-oil emulsion separation are not well known. Herein, we report the synthesis of superhydrophobic hollow hemispherical MXene (HSMX) via a sacrificial template approach by thermal annealing of MXene-wrapped cationic polystyrene spheres (CPS@MXene). The corresponding HSMX-based 3D membrane was subsequently prepared by filtration. The application of the membrane for the separation of emulsifier-stabilized water-in-oil emulsions was investigated, and a possible separation mechanism was also proposed.

## 2. Materials and Methods

### 2.1. Materials

Titanium aluminum carbide (Ti_3_AlC_2_) was bought from the Hello Nano Technology Co. Ltd., Changchun, China. Styrene, methanol, ethanol, hydrochloric acid (HCl, 36.0%–38.0% in H_2_O), and methylene blue trihydrate (MB, C_16_H_18_ClN_3_S·3H_2_O) were purchased from Sinopharm Chemical Reagent Co. Ltd., Shanghai, China. 2,2′-Azobis (2-methylpropionitrile) (AIBN, ≥98%) and methacrylatoethyl trimethyl ammonium chloride (DMC, 75% in H_2_O) were provided by MACKLIN, Shanghai, China. Paraffin liquid and Span 80 were provided by Tianjin Damao Chemical Reagent Factory and Tianjin Guangfu Fine Chemical Research Institute, Tianjin, China, respectively. Dichloromethane was provided by Chinasun Specialty Products Co. Ltd., Changshu, China. All chemicals were of analytic reagent grade and were used as received.

### 2.2. Synthesis of Cationic Polystyrene Spheres

Cationic polystyrene spheres (CPS) were prepared via dispersion polymerization. Briefly, 0.1 mL of DMC as a polymeric stabilizer was added into a mixed solution consisting of 36 mL of methanol and 4 mL of deionized water (DI) in a flask, and then mechanical stirred for 10 min under a nitrogen atmosphere. A mixture of 4 mL of styrene and 0.036 g of AIBN as the initiator was then added dropwise into the above solution and the polymerization was conducted at 80 °C for 6 h. The obtained product was washed successively with ethanol and DI 6 times by repeated centrifugation and finally redispersed in 20 mL DI to obtain an aqueous dispersion of CPS (50 mg/mL).

The number average molecular weight M_n_, the weight average molecular weight M_w_, and the polydispersity of the synthesized CPS were 195,130 g/mol, 515,553 g/mol, and 2.642, respectively, as determined by gel permeation chromatography (PL-GPC50, Polymer Laboratories Ltd., Palo Alto, CA, USA). The zeta potential of CPS was 5.53 mV, which was measured using a Grain Size Analysis-Zeta Potentiometer (ZS90, Malvern Instruments Ltd., Malvern, UK).

### 2.3. Preparation of Hollow Hemispherical MXene (HSMX)

**Scheme 1:**
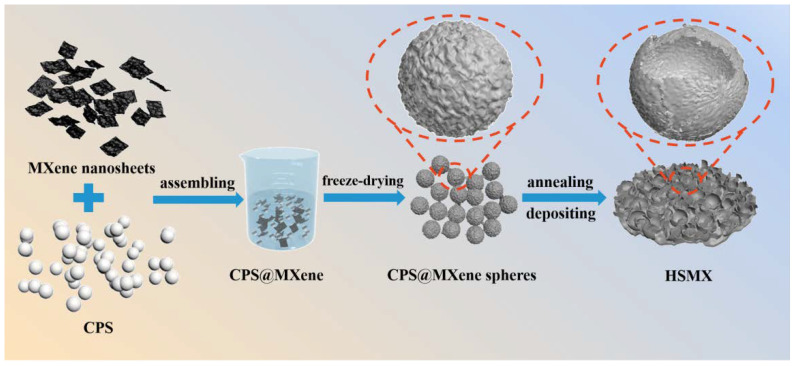
Synthesis process for hollow hemispherical MXene (HSMX) and the corresponding 3D membrane.

The aqueous suspension of exfoliated Ti_3_C_2_T_x_-MXene nanosheets was prepared according to the detailed procedure of Wang et al. [37]. The zeta potential of the obtained MXene was −7.29 mV. The MXene-wrapped CPS (CPS@MXene) spheres were first prepared based on the electrostatic interaction between CPS and MXene. A typical synthesis procedure for CPS@MXene spheres at a CPS to MXene mass ratio of 10:1 was as follows: 20 mL of CPS dispersion was mixed with 25 mL of MXene suspension (4 mg/mL) and magnetically stirred for 2 h at room temperature. Subsequently, the mixture was transferred into a lyophilizer and frozen at −56 °C for 24 h. The CPS@MXene spheres were then obtained via vacuum freeze-dried for 72 h. The hollow hemispherical MXene (HSMX) was finally obtained after thermal annealing of CPS@MXene spheres at 410 °C for 2 h under a nitrogen flow. A schematic of the preparation procedure is illustrated in Scheme 1.

The zeta potentials of CPS@MXene and the corresponding HSMX were −3.85 and −34.5 mV, respectively, suggesting that they had a negatively charged surface.

### 2.4. Preparation of 3D Superhydrophobic HSMX Membrane

An HSMX dispersion was prepared by adding 15 mg of HSMX into 2 mL of ethanol, followed by ultrasonic agitation for 1 min. The 3D HSMX membranes were obtained by depositing HSMX onto porous polyvinylidene fluoride (PVDF) substrates with an average pore size of 0.22 μm by vacuum filtration.

### 2.5. Preparation of Water-in-Oil Emulsions

Two types of water-in-oil emulsions containing droplets of different sizes were prepared. An ordinary water-in-paraffin liquid emulsion (emulsion-I) was prepared via vigorously stirring a mixture of paraffin liquid and MB solution (80 mg/L) (100:1, volume/volume, the corresponding water content was 8667 ppm, which was measured using a Karl Fischer moisture titrator) for 3 h. The other type of water-in-paraffin liquid emulsion (emulsion-II), with smaller droplets that were stabilized by an emulsifier, were prepared by vigorously stirring 100 mL of paraffin liquid and 0.85 g of Span 80 for 10 min, followed by mixing with 1 and 2 mL of MB solution for 6 h, giving a corresponding water content of 8889 and 14286 ppm, respectively; these were denoted as emulsion-II (1) and emulsion-II (2).

### 2.6. Characterization

The morphology of the samples was observed using a SU8010 field-emission scanning electron microscope (FE-SEM) with an acceleration voltage of 15 kV. TEM images were obtained using a JEOL JEM 2100PLUS transmission electron microscope. Attenuated total reflection Fourier-transform infrared (ATR-FTIR) spectra were recorded by a Nicolet spectrometer (Thermo Fisher, Waltham, MA, USA). The X-ray diffraction (XRD) patterns were measured on an X-ray diffractometer (Rigaku Co., Tokyo, Japan) with Cu K_α_ radiation (λ = 0.1542 nm). Mercury intrusion porosimetry (MIP) measurements were conducted on a Micromeritics AutoPore IV 9500. Thermogravimetric analysis (TGA) was performed on a TG 209 F1 thermoanalyzer instrument. A sample of 4–10 mg was heated from room temperature to 600 °C at a heating rate of 10 °C/min under a nitrogen flow. Atomic force microscopy (AFM) was conducted on a DI Multimode V scanning probe microscope (Bruker, Karlsruher, Germany) in tapping mode. The water contact angle (WCA) was measured at room temperature via a contact angle meter (DSA100, Kruss, Hamburg, Germany). The droplet volume was approximately 3 µL for static WCA tests and 12 μL for rolling angle tests.

The water-in-oil emulsion separation experiments were carried out by adding emulsions onto the 3D HSMX membrane using a filtration setup under 0.4–0.6 bar vacuum pressure. Optical microscope images of emulsions and filtrates were observed by a polarized light microscope (AxioScope.A1), and their corresponding droplet size distributions were measured by dynamic light scattering (Malvern Zetasizer Nano ZS, Hamburg, Germany). The separation *Flux* was expressed by the formula:

(1) $$Flux=\frac{V}{SPt}$$

where *V* is the volume of filtered oil (L), *S* is the valid surface area of membrane in contact with the emulsion (m^2^), *P* is the additional pressure (bar), and *t* is the testing time (h). The separation efficiency (SE) was calculated as follows:

(2) $$SE=(1-\frac{C_{F}}{C_{E}})\times 100\%$$

where *C_E_* and *C_F_* represent the water content of the emulsions and their corresponding filtrates, respectively. The water content was measured using a Karl Fischer moisture titrator (870 KF Titrino plus).

## 3. Results

### 3.1. Characterization of HSMX

**Figure 1:**
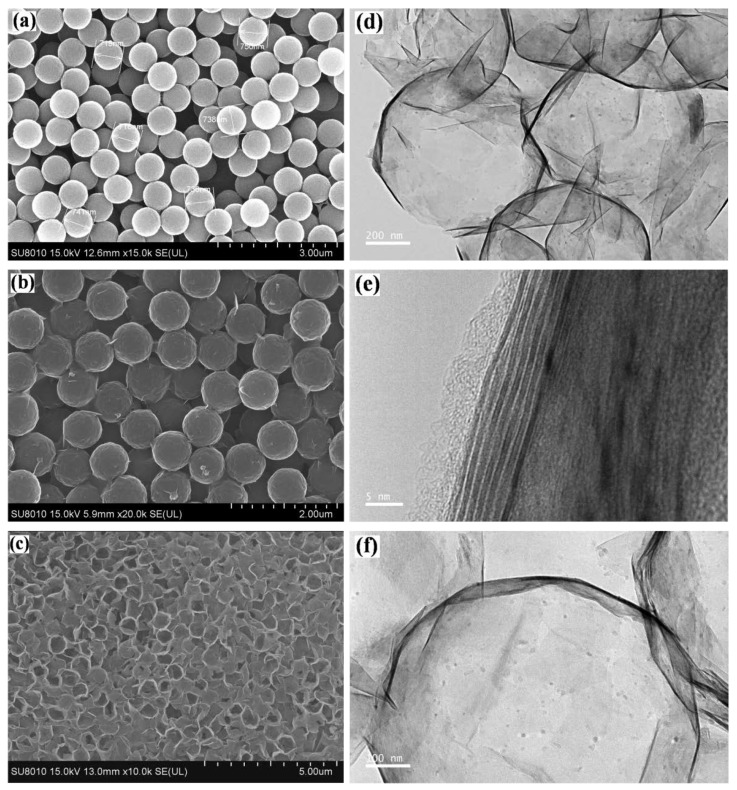
SEM images of (**a**) CPS and (**b**) CPS@MXene spheres, and (**c**) the as-prepared HSMX. (**d**–**f**) TEM images of HSMX and its spherical wall.

The morphology and microstructure of CPS@MXene spheres and the corresponding HSMX were revealed by SEM and TEM images, as shown in Figure 1. The CPS@MXene spheres were 785 nm in diameter with a much rougher surface, owing to the wrapping of MXene nanosheets, in comparison with that of the 735 nm CPS spheres (see Figure 1a,b). After thermally evaporating CPS from CPS@MXene spheres, a honeycomb-like macroporous architecture composed of mostly hollow hemispherical MXene (HSMX) was obtained (see Figure 1c). The TEM image in Figure 1d demonstrates that these hollow hemispheres have good electron transparency, and the flexible MXene nanosheets are curled and stacked on top of each other. As measured directly from Figure 1e, the wall thickness of the hollow hemispheres is 8–10 nm, which comprises several MXene nanosheets, where the average thickness of an individual MXene nanosheet and the distance between them are 0.81 and 0.40 nm, respectively. Notably, many nanoparticles, perhaps carbon nanoparticles (CNPs), were found to be distributed on the surface and inserted in the interlayers of MXene (see Figure 1f).

**Figure 2:**
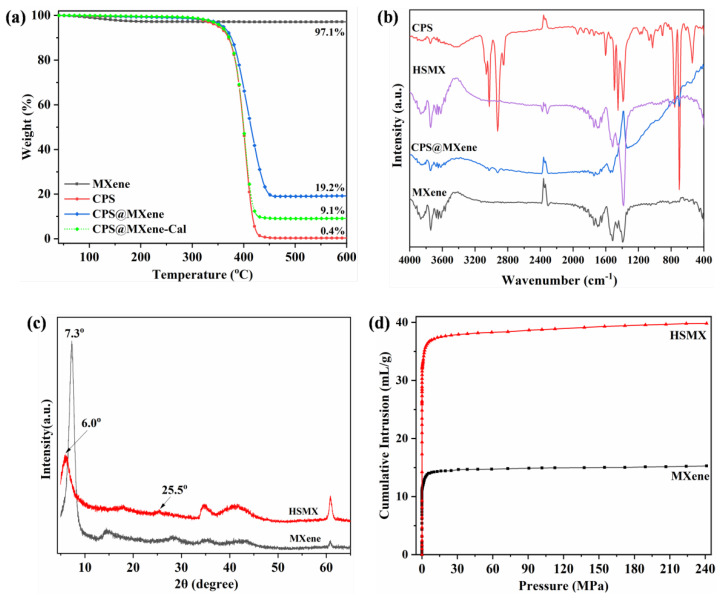
(**a**) TGA curves of MXene, CPS, and CPS@MXene. MXene and HSMX characterized by (**b**) FTIR, (**c**) XRD patterns, and (**d**) plots of MIP cumulative intrusion versus pressure.

The composition and structure of the as-prepared HSMX were investigated by TGA, FTIR, and XRD. Figure 2a shows the TGA curves of MXene, CPS, and CPS@MXene spheres. MXene exhibits high thermal stability with slight mass loss due to the removal of physically absorbed water and unstable groups on its surface [38]. The CPS degraded almost completely at temperatures higher than 400 °C and left only 0.4% residual char at 600 °C. CPS@MXene showed a remarkable enhancement of thermal stability and a greater char yield when the temperature was increased to 400 °C (see Table 1). A comparison of the experimental char yields (Char) with the theoretical data (Cal-char) of CPS@MXene calculated by linear combinations of the experimental TGA curves of the single components, CPS and MXene, is presented. It is apparent that the CPS@MXene showed a remarkable 111.0% enhancement in char experimentally compared with the calculated amount (CPS@MXene-Cal). The results clearly support that the MXene wrapping of CPS spheres can efficiently promote the carbonization of the degradation products from CPS, producing more char.

Figure 2b shows the FTIR spectra of CPS, MXene, CPS@MXene, and HSMX. CPS shows characteristic absorption bands in the ranges of 700–756, 1450–1500, and 2800–3200 cm^−1^, corresponding to the out-of-plane C–H bending bands, C–C stretching vibrations in the aromatic ring, and the C–H stretching vibration of –(CH_2_)_n_–, respectively [39,40]. MXene shows characteristic peaks at 1380, 1520, and 3740 cm^−1^, respectively, which were assigned to the stretching vibrations of –OH, –C=O, and –OH, respectively, reflecting the abundance of termini of the Ti_3_C_2_T_x_-MXene nanosheets [41,42]. It is noteworthy that the intensities of characteristic frequencies, in particular, the –(CH_2_)_n_– vibration in the 2800–3200 cm^−1^ range of CPS in CPS@MXene, decreased significantly, indicating the successful wrapping of MXene on the surface of CPS. HSMX has a similar FTIR spectrum to MXene, accompanied with the disappearance of the characteristic bands of CPS, which verified that the CPS templates were completely removed during the thermal annealing process. The XRD patterns shown in Figure 2c revealed that the characteristic (002) peak of MXene initially at 2θ = 7.3° shifted to a lower angle at 6.0° in HSMX, which indicated that some intercalation into the interlayer occurred and therefore increased the d-spacing by approximately 0.26 nm. It should also be noted that a broadened, low-intensity peak appeared at 25.5° in HSMX, which was assigned to the (002) plane of carbon structure with a d-spacing of 0.35 nm. The XRD observations are in good agreement with the TEM images, and confirm the insertion of CNPs into the interlayer of MXene.

Based on the results above, it can be reasonably concluded that the spherical MXene shells promote the char formation (mainly from CNPs) of CPS@MXene. It is postulated that, during the thermal annealing process, HSMX can effectively retain the pyrolysis volatiles of CPS so that they have enough time to diffuse into the interlayer of MXene. Some interactions between those volatiles and MXene nanosheets may subsequently occur to induce reactions such as crosslinking and cyclization for the “in-situ” generation of char [43], even from CNPs.

### 3.2. Superhydrophobic Performance

The porous structure and surface morphology are both important factors that determine the surface wetting behavior of membranes and the potential to achieve good hydrophobic or hydrophilic properties. The variation in composition, in particular the unexpected product of CNPs, may be an important factor affecting the porous structure of HSMX. This hypothesis was verified by comparing the MIP cumulative intrusion curves between 2D MXene and 3D HSMX, as shown in Figure 2d, with the related parameters listed in Table 2. It appears that as the insertion of CNPs increases the interlayer distance of MXene and weakens the interactions between layers, the 3D interconnected honeycomb-like ar-chitecture endows HSMX with a higher porosity (97.8%), total pore volume (39.8 mL/g), and total pore area (703.1 m^2^/g) than MXene, but a lower packing density (0.025 g/mL).

**Figure 3:**
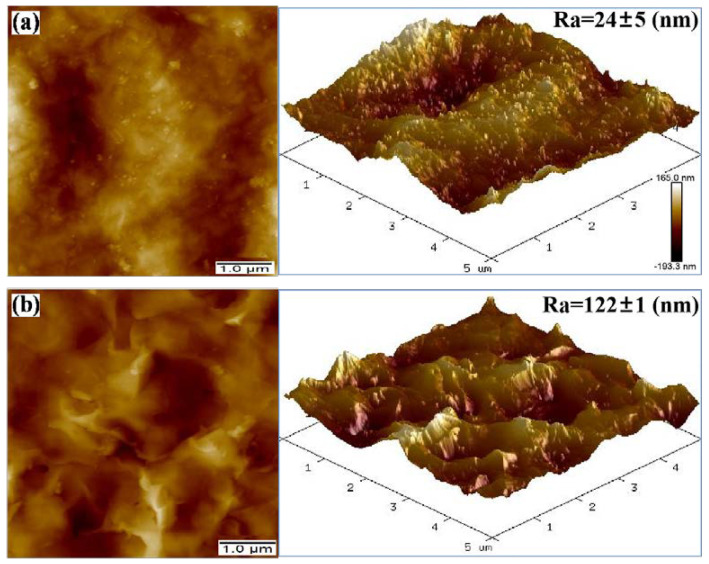
AFM images of (**a**) MXene and (**b**) HSMX membranes.

This unique porous structure is expected to give the corresponding HSMX membrane a complex microscopic surface morphology and to increase its surface roughness, which is strongly required to achieve superwettability, such as superhydrophobicity or superhydrophilicity [44]. AFM imaging, as shown in Figure 3, was applied to distinguish the surface characteristics of 2D MXene and 3D HSMX membranes. The value of the arithmetical mean deviation of the profile (R_a_) is a measure of the 3D contour of a sample surface and represents the surface roughness. The AFM images of the two kinds of membranes present distinct surface microstructures. The surface of the 2D compact MXene membrane was much smoother, with a low R_a_ value of 24 nm (see Figure 3a), whereas the stacking of hollow hemispheres promoted the formation of a highly undulating macroporous surface and significantly increased the surface roughness, resulting in a high value of R_a_ of 122 nm in HSMX (see Figure 3b).

**Figure 4:**
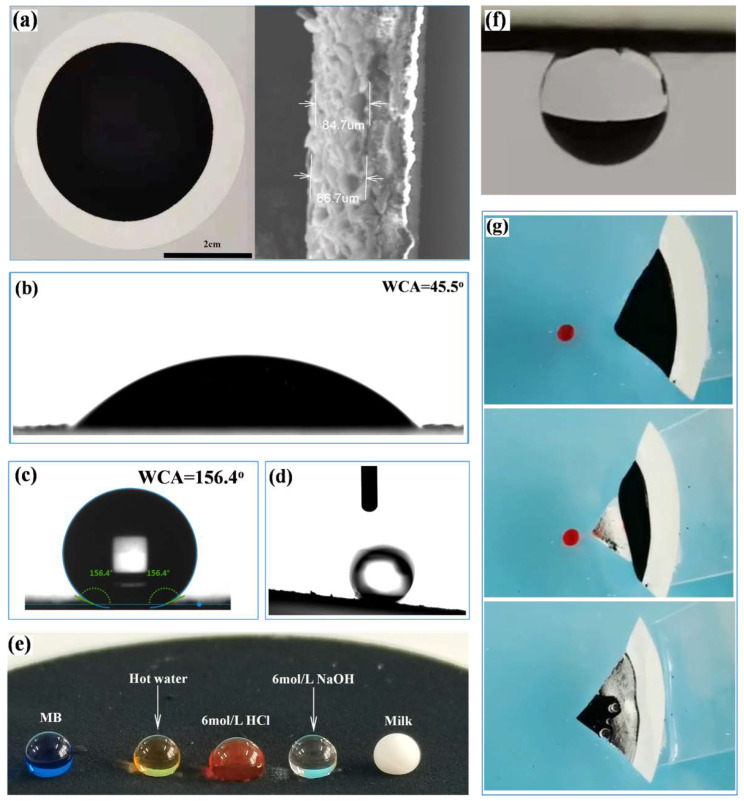
(**a**) Photograph of HSMX membrane and SEM image of its cross-section. Water contact angles of (**b**) MXene and (**c**) HSMX membranes, (**d**) rolling angle of water droplet on HSMX membrane, (**e**) different droplets on HSMX membrane, (**f**) water droplet hanging upside down on HSMX membrane. (**g**) Photographs of separating dichloromethane from the water-in-oil system using HSMX membrane and the silver mirror phenomenon.

The surface wetting behavior of the membrane is critical to practical applications, and the tests were performed on the HSMX membranes with a thickness of approximately 85 μm and a density of 138.7 ± 7.5 mg/cm^3^ (Figure 4a). The compact 2D MXene membrane exhibits good hydrophilicity with a water contact angle (WCA) of 45.5° (see Figure 4b). In stark contrast, the 3D HSMX unexpectedly exhibits hydrophobic performance with the WCA increasing to 156.4° (see Figure 4c). Additionally, the rolling angle of HSMX membrane was as small as 6° (see Figure 4d). These results suggest that the HSMX membrane meets the requirements of a superhydrophobic surface. It is clear that the conversion from hydrophilic 2D MXene to superhydrophobic 3D HSMX occurs mainly because the porous structure changes the surface morphology of HSMX and significantly increases the surface roughness, thereby improving the wettability to the extreme state of superhydrophobicity.

**Scheme 2:**
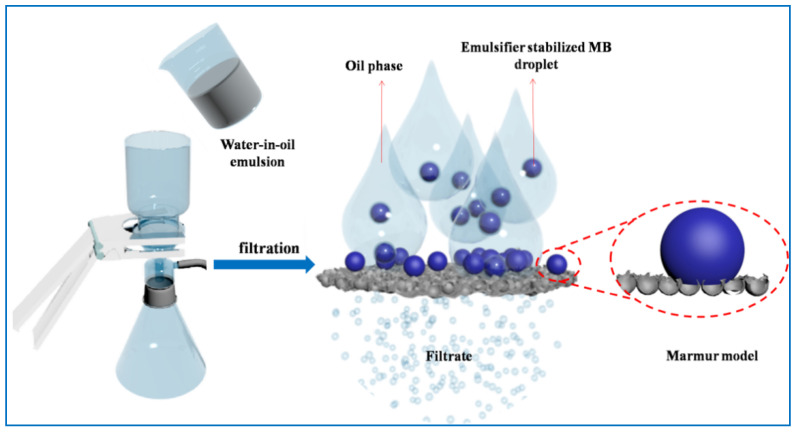
Possible separation mechanism of water-in-oil emulsions on the HSMX-based membrane.

Furthermore, the HSMX membrane exhibits satisfactory hydrophobicity and good self-cleaning properties in harsh conductions, such as strong acid and alkali. This was demonstrated as, despite the addition of various droplets, including MB, hot water (70 °C), HCl (6 mol/L), KOH (6 mol/L), and milk, to the membranes, they retained spherical structure with a WCA larger than 90° (see Figure 4e), confirming the feasibility of the membrane for industrial and biological applications. Interestingly, a water droplet hanging upside down on the HSMX membrane will not drip (see Figure 4f), which may indicate that the contact mode of the water droplet on the membrane surface conforms to the Marmur model [45,46]. Namely, the water droplet partially enters the concave–convex grooves formed by the 3D HSMX nanofolds, as depicted in Scheme 2. The HSMX membrane also exhibited the ability for rapid separation of dichloromethane underwater; a silver mirror phenomenon [41] appeared at the interface between HSMX and water, accompanied by the repellent bubbles adhering to the surface of HSMX (see Figure 4g). It is believed that this phenomenon is not only related to the strong repulsion of the superhydrophobic membrane to water, but also related to the Marmur model.

### 3.3. Water-in-Oil Emulsion Separation Performance

**Figure 5:**
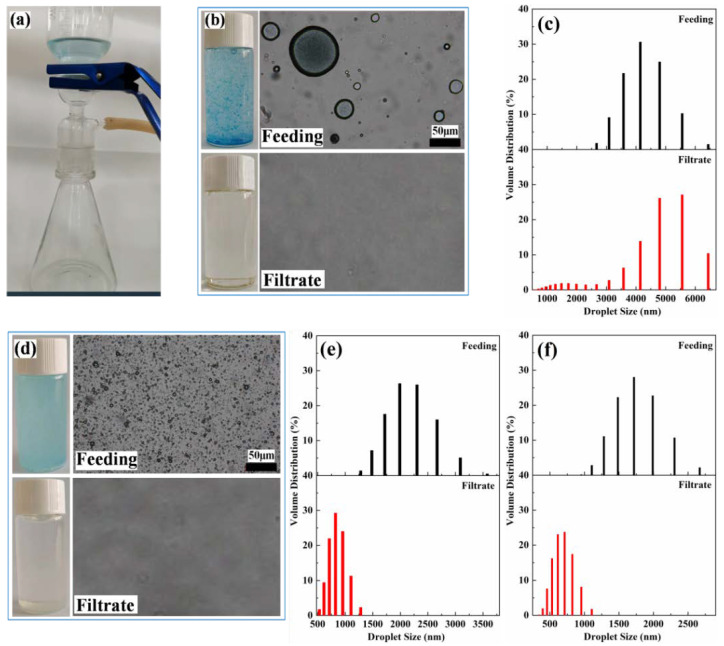
(**a**) Digital photographs of water-in-oil emulsion separation apparatus, and separation results of (**b**,**c**) emulsion-I, (**d**,**e**) emulsion-II (1), and (**f**) emulsion-II (2).

The separation performance of the HSMX membrane for water-in-paraffin liquid emulsions was examined using a separation setup (see Figure 5a). It was observed that the micron-sized water droplets with a broad polydispersity were dispersed in the unstable emulsion-I, but few droplets could be observed in the filtrate (see Figure 5b,c). Correspondingly, the membrane had a high separation efficiency (SE) of 97.6%, and the residual water content in the filtrates was as low as 209 ppm. Meanwhile, the average droplet sizes decreased from 4243 ± 770 nm in the feed to 1521 ± 464 nm in the filtrate, as listed in Table 3.

The better the stability of an emulsion, the more difficult the de-emulsification and separation. As shown in Figure 5(d,e), the emulsion-II (1), with a smaller droplet size of 2151 ± 432 nm, had better homogeneity and stability. After separation, the droplet sizes were decreased to 849 ± 160 nm. The emulsion-II (2) followed a similar trend, with a decrease in droplet sizes from 1745 ± 343 to 681 ± 153 nm (see Figure 5f). Meanwhile, the membrane maintained effective separation of the stable emulsions and the SE was 97.3% and 96.5% on emulsion-II (1) and (2), respectively. However, the water content in the filtrates was slightly increased, to 240 and 500 ppm, respectively, whereas the separation *Flux* for emulsion-II (1) and (2) was reduced to 159.4 and 86.3 L/m^2^·h·bar, respectively, compared with 354.6 L/m^2^·h·bar for emulsion-I. The reduction in separation performance, in particular for emulsion-II (2), may be related to the water content and droplet size distributions of the feeding emulsion. That is, the release of a large number of smaller sized water droplets from water-in-oil emulsion droplets is more likely to form a dense water film on the surface of HSMX, which prevents the oil phase from contacting HSMX and increases the filtration resistance, thus reducing the separation performance of the HSMX membrane.

The separation mechanism of water-in-oil emulsion on HSMX membrane was proposed, as illustrated in Scheme 2. When in contact with the emulsion, the oil phase can be quickly captured and separated through the membrane. Importantly, the nanoscale spherical walls of HSMX can serve as sharp razors that effectively demulsify the stabilized water–surfactant–paraffin emulsion droplets, and then separate the water phase so as to realize the emulsion separation. However, the HSMX membrane is prone to detaching from the surface of a PVDF substrate and to contamination owing to its inherent oleophilic properties, which makes the long-term stability and separation recyclability unsatisfactory. More work is needed to improve the stability of HSMX on PVDF surfaces.

## 4. Conclusions

In summary, this work demonstrates the fabrication of hollow hemispherical MXene (HSMX) by thermal annealing of CPS@MXene spheres via a sacrificial template approach. The as-prepared HSMX not only imparts the corresponding 3D membrane with superhydrophobicity and good antipollution performance in harsh conductions, but also confers a high separation efficiency (above 96.5%) for the separation of Span 80-stabilized water-in-paraffin liquid emulsions, with a separation *Flux* above 86.3 L/m^2^·h·bar. Detailed analyses indicate that the spherical MXene shells of CPS@MXene promote the catalytic carbonization of CPS into CNPs. In particular, CNPs confer major a benefit to the construction of a unique macroporous structure of HSMX, which helps to induce the formation of a complex microscopic surface morphology and significantly improve the surface roughness, thereby transforming the hydrophilic surface of 2D MXene to the superhydrophobic surface of 3D HSMX. The progress on the synthesis of superhydrophobic HSMX is of great importance for expanding the applications of MXene-based materials, and has promising prospects for environmental and catalytic carbonization applications.

## Figures and Tables

**Table 1:** Thermal properties of CPS@MXene spheres under nitrogen flow.

Sample	TGA				
	T_10%_ (°C)	T_max_ (°C)	Char (%, 600 °C)	Cal-Char (%, 600 °C)	Enhancement in Char Compared to Cal-Char (%)
CPS	370.2	403.6	0.4	NA	NA
MXene	NA	560.6	97.1	NA	NA
CPS@MXene	375.5	411.5	19.2	9.1	111.0

T_10%_, temperature at which 10% degradation occurs; T_max_, temperature obtained from derivative TG (DTG) curves at which the maximum mass loss rate occurs.

**Table 2:** Comparison of MIP parameters of MXene and HSMX.

Sample	Porosity (%)	Packing Density (g/mL)	Average Pore Diameter (nm)	Total Pore Volume (mL/g)	Total Pore Area (m^2^/g)
MXene	95.3	0.062	1355.3	15.3	294.3
HSMX	97.8	0.025	1431.2	39.8	703.1

**Table 3:** Separation performance of the HSMX membrane to separate water-in-oil emulsions.

Emulsions	Droplet Size (nm)				Water Content in Filtrate (ppm)	Separation Efficiency (%)	Separation Flux (L/m^2^·h·bar)
	Feeding		Filtrate				
	Distribution	Average	Distribution	Average			
Emulsion-Ⅰ	2600–6500	4243 ± 770	530–6500	1521 ± 464	208	97.6	354.6
Emulsion-Ⅱ (1)	1200–3600	2151 ± 432	530–1300	849 ± 160	240	97.3	159.4
Emulsion-Ⅱ (2)	950–2700	1745 ± 343	340–1200	681 ± 153	500	96.5	86.3

## Data Availability

The data presented in this study are available on request from the corresponding author.
